# Evolution of Grain Interfaces in Annealed Duplex Stainless Steel after Parallel Cross Rolling and Direct Rolling

**DOI:** 10.3390/ma11050816

**Published:** 2018-05-16

**Authors:** Ming Wang, Haoqing Li, Yujing Tian, Hong Guo, Xiaoying Fang, Yuebin Guo

**Affiliations:** 1 Institute for Advanced Manufacturing, Shandong University of Technology, Zibo 255000, China; 18369904566@163.com (M.W.); abkkl8ba@126.com (H.L.); 17852036226@163.com (Y.T.); yguo@eng.ua.edu (Y.G.); 2 School of Mechanical Engineering, Shandong University of Technology, Zibo 255000, China; 3 Center of Testing and Analysis, Shandong University of Technology, Zibo 255000, China; guohong0831@126.com; 4 Department of Mechanical Engineering, The University of Alabama, Tuscaloosa, AL 35487, USA

**Keywords:** grain interface, twin boundary, cross rolling, duplex stainless steel

## Abstract

Changes in various grain interfaces, including the grain boundary and phase boundary, are a strong indication of microstructural changes, particularly ultra-fined grains achieved by large strain deformation and subsequent annealing. After direct rolling and cross rolling with the same strain of ε = 2, the distributions of the interfaces in annealed UNS S32304 duplex stainless steel were investigated using electron backscatter diffraction (EBSD) in this study. The ferrite experienced continued recovery, and a high density of low-angle grain boundaries (LAGBs) was produced. The percentage and number of twin boundaries (TBs) and LAGBs varied within the austenite. TBs were frequently found within austenite, showing a deviation from the Kurdjumov-Sachs (K-S) orientation relationship (OR) with ferrite matrix. However, LAGBs usually occur in austenite, with the K-S OR in the ferrite matrix. LAGBs were prevalent in the precipitated austenite grains, and therefore a strong texture was introduced in the cross-rolled and annealed samples, in which the precipitated austenite readily maintained the K-S OR in the ferrite matrix. By contrast, more TBs and a less robust texture were found in the precipitated austenite in direct-rolled and annealed samples, deviating from the K-S OR.

## 1. Introduction

Duplex stainless steels (DSSs) are widely used in the petroleum, chemical, and nuclear power industries due to their excellent mechanical properties and corrosion resistance [1,2,3]. Microstructural features such as grain size [4], phase distribution [5], grain texture [6], and in particular, the interface character distribution, are very influential in the material performance [7,8]. There exist two categories of interface in DSSs with constituent phases of ferrite (α) and austenite (γ): a homophase interface including a grain boundary within the respective α and γ, and a heterophase interface, also known as the phase boundary, between the γ and α grains. DSSs inherently possess a refined microstructure due to the presence of phase boundaries when subjected to thermal mechanical treatment, and therefore exhibit better mechanical properties and corrosion resistance when two constituent phases are well-distributed, as compared to single-phase alloys [9,10]. However, the clustering of homophase grains frequently occurs, which leads to grain coarsening to some extent during thermal cycles, ultimately producing a negative effect on the material performance. For a favorable microstructure in duplex alloys such as DSSs, the α and γ phases should have relatively similar proportions and uniform distributions. It is expected that the percentage of the phase boundary in the total interfaces in DSSs is a direct measure of the degree of homogeneity of phase distribution for a given phase volume proportion. The higher the percentage of phase boundary, the more homogenous the duplex phase distribution. It is well recognized that the resulting microstructure mainly depends on the thermal mechanical processing (TMP) history. The conventionally processed DSS has a typical band microstructure of alternating elongated α and γ grains layers in the rolling direction. To interrupt the band morphology and achieve a homogenous duplex microstructure, introducing the initial microstructure of single full ferrite and then employing large strain and annealing has been proven to be an effective method [11,12]. However, it is also found that clustering of homophase grains, especially γ grains, emerges at a relatively small scale, although the long band microstructure is interrupted over a large scale.

The characters of grain boundary and phase boundary have been investigated based on the boundary misorientation between neighboring grains using transmission electron microscopy (TEM) and electron backscatter diffraction (EBSD) techniques in previous studies [13,14]. It was found for the band duplex microstructure, a large fraction of high angle grain boundaries was present in the ferrite and twin boundaries (TBs) in austenite, and more phase boundaries deviated from the Kurdjumov-Sachs (K-S) orientation relationship. By comparison, a high fraction of low-angle grain boundaries (LAGBs) in ferrite and TBs coexist in austenite in the uniformly distributed duplex microstructure, and the amount of phase boundary meeting Kurdjumov-Sachs (K-S) orientation relationship (OR) increases. A large discrepancy lies in the different types of microstructural evolution involved [13]. However, the correlative dependence of the grain boundary character and phase boundary character has not yet been clarified. In the current study, the effect of TMP on the characters of the grain boundary and phase boundary are investigated first, and then the correlative dependence of the evolution of the grain boundary and the phase boundary character in terms of misorientation is discussed in detail.

## 2. Materials and Methods

**Figure 1:**
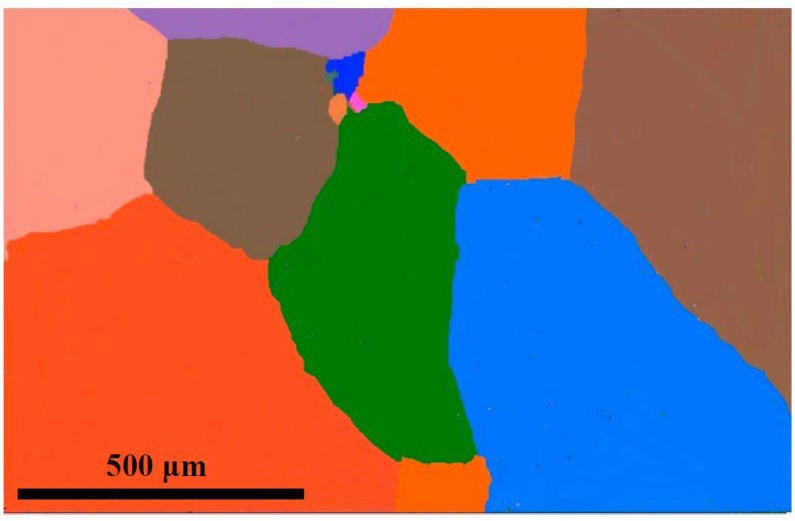
An electron backscatter diffraction (EBSD)-reconstructed orientation image microscopy (OIM) map of the initial sample.

**Figure 2:**
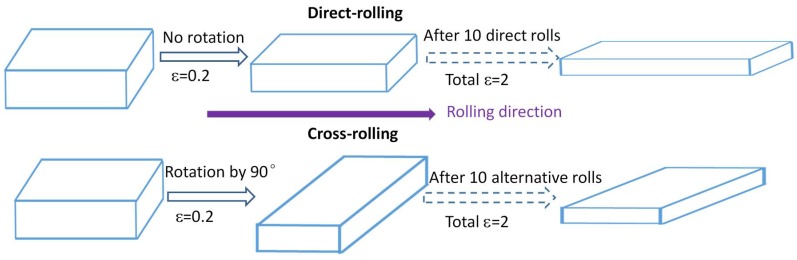
Schematic of cross-rolling and direct-rolling.

A typical UNS S32304 duplex stainless steel (DSS) was used in this study. The chemical compositions were 23.77 wt % Cr, 4.18% Ni, 1.2 wt % Mn, 0.011 wt % C, 0.002 wt % S, 0.026 wt % P, 0.102 wt % N, 0.5 wt % Si, 0.21 wt % Cu, and 0.17 wt % Mo. The samples were cut from the hot-rolled DSS plate by wire-electrode cutting, and then solution-heat-treated (SHT) for 30 min at 1573 K to obtain a single supersaturated ferritic microstructure with an average grain size of 500 μm as shown in Figure 1. The SHT samples were divided into three groups for further TMP treatment. The first was annealed at 1323 K for 2 min without pre-deformation and the austenite was precipitated directly from the supersaturated ferrite. The second sample group was subjected to 10 rolls of single-directional rolling in sequence with a total true strain ε of 2 (0.2 strain increment/rolling) under the rolling speed of 416 mm/s. The third sample group was subjected to cross-rolling, which involved the same total true strain of 2 (0.2 strain increment/rolling) for 10 rolls but with an alternating perpendicular rolling pattern [15] under the same rolling speed. The rolling plane was same, but the initial and final thicknesses of the rolled samples were 15 mm and 2 mm, respectively. The rolling operations were conducted at room temperature and schematically mapped in Figure 2. The samples with dimensions of 12 mm (RD) × 10 mm (TD) × 2 mm (ND) were cut from the two ultimately rolled plates and then subsequently annealed at 1323 K for 2 min. The above three samples were named A, B, and C, respectively.

EBSD mapping was conducted on the rolling plane (RD × TD) in the samples as processed. The examined surface was first mechanically polished using standard emery papers of up to grade 2000, followed by electro-polishing in a solution of HClO_4_:CH_3_COOH = 20:80 (volume fraction) under 30 V for 20 s. The EBSD system was an HKL-Channel 5 attached to a Sirion-200 field emission scanning electron microscope (FESEM, FEI, Hillsboro, OR, USA). The step size of EBSD mapping was 0.2 μm. The zero solution pixels with difficulty in indexing due to the grain boundary, phase boundary, contamination, etc. were reduced according to the clean-up procedure within the framework of HKL Channel 5 software. Their orientations were assigned based on at least four neighboring pixels with a common orientation. The indexed rates of Kikuchi patterns were better than 90%. The grain orientation of the precipitated austenite in three samples was analyzed in the {001} pole figure (PF) space. Considering that austenite typically holds a K-S OR with the ferrite matrix, the <001> directions of the exact 24 K-S variants of austenite from its parent ferrite were also represented in PF space using the PTClab software v1.1 developed by Gu [16]. There are typically two types of interfaces: grain boundaries (GBs) and phase boundaries (PBs). Here, an individual grain was defined as a region being completely bounded by phase boundaries and/or grain boundaries that have a misorientation angle larger than a critical value of 2°. Misorientation in the form of angle/axis pairs (θ/<uvw>) for GBs and orientation relationships (ORs) in the form of parallelism conditions applying to the crystallographic planes and direction ({hkl}γ//{hkl}α, {uvw}γ//{uvw}α) for PB were employed to evaluate the orientation difference between the abutting grains.

## 3. Results and Discussion

### 3.1. Microstructure after TMP

**Figure 3:**
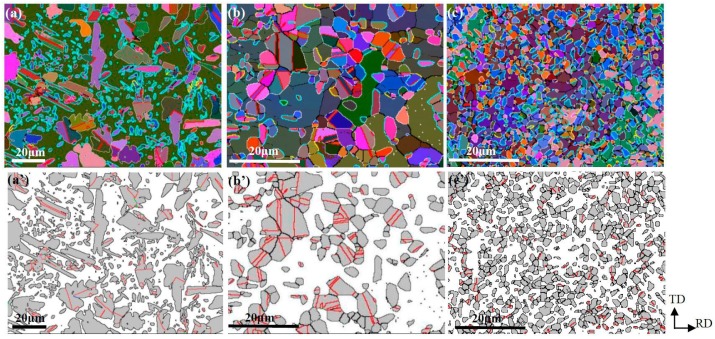
The EBSD-reconstructed microstructure of samples A (**a**,**a’**), B (**b**,**b’**) and C (**c**,**c’**). (**a**–**c**) correspond to OIMs based on three Euler angles, where the bright blue lines represent the phase boundary with a Kurdjumov-Sachs (K-S) orientation relationship (OR) within 3.5°; (**a’**–**c’**) represent the phase distribution, where the gray and white regions correspond to γ and α phases, respectively, with the red lines representing twin boundaries (TBs) in γ.

**Figure 4:**
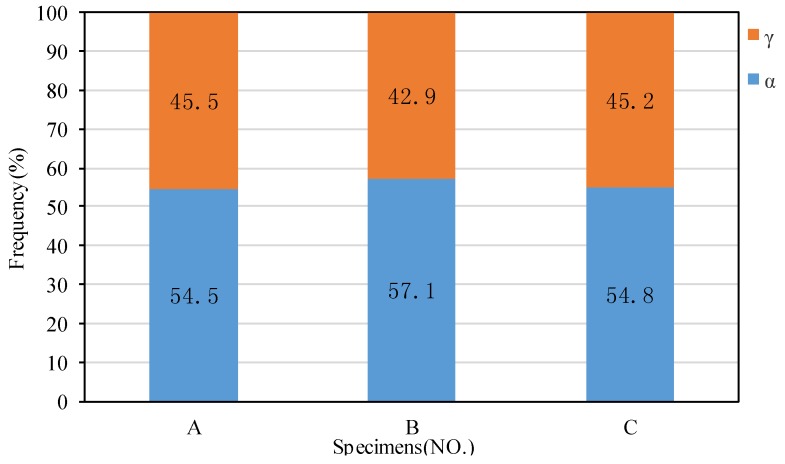
The fractions of γ and α in samples A, B, and C.

Figure 3 shows the EBSD-reconstructed microstructure and phase distribution in samples A, B, and C. The area fractions of γ and α are also given in Figure 4, indicating the area ratios of Aγ:Aα were close to 1:1 for all three samples. It is obvious that a nearly equivalent amount of γ phase precipitated from the undeformed supersaturated ferrite (sample A) and deformed supersaturated ferrite (samples B and C) upon annealing for 2 min at 1323 K. In addition, the band microstructure morphology was not present due to the high-temperature solid solution treatment at 1573 K prior to TMP. Considering the initial coarse ferritic matrix, the current paper mainly focuses on the precipitated γ within ferritic grains, as the grain boundary γ precipitation is beyond the scope of this work.

A large microstructural discrepancy exists in γ precipitation among three samples. First, the most refined and uniform duplex grains are obtained in sample C. The average size of both the α and γ grains is nearly the same at about 2 μm. The α grains are larger than the γ grains in sample B, for which the average sizes are 17 μm and 5 μm, respectively. A bimodal grain structure emerges for austenite directly precipitated from the coarse ferrite in sample A. The enlarged and fined grains have an average grain size of 18 μm and 3 μm, respectively. Second, the γ grain appearance is quite different from sample to sample. The large γ grains exhibited rectangular shape with an aspect ratio of over 3, whereas the small grains appear as equiaxed ones in sample A. By comparison, equiaxed γ grains are present in both samples B and C. Third, the γ grain-clustering phenomena are more pronounced in sample B than in samples A and C. The clustered grains tend to coarsen due to the absence of phase boundaries in the local region. The difference can be explained as follows: the pre-deformed supersaturated ferrite underwent recovery, and the dislocation was rearranged into the low energy dislocation wall, which acted as nucleation site for γ precipitation, and therefore more equiaxed and uniform γ grains were found in samples B and C. However, the stronger strain anisotropy resulting from single directional rolling in sample B contributed to the clustering and growth of austenite compared to the cross-rolled sample C during annealing. If the total interfaces (including PBs and GBs) are normalized to 100, the number ratios of PBs and GBs for samples B and C are approximately 55:45 and 65:35, respectively. It is indicated that less clustering of γ grains appears in sample C.

### 3.2. Grain Boundary Character Distribution (GBCD)

**Figure 5:**
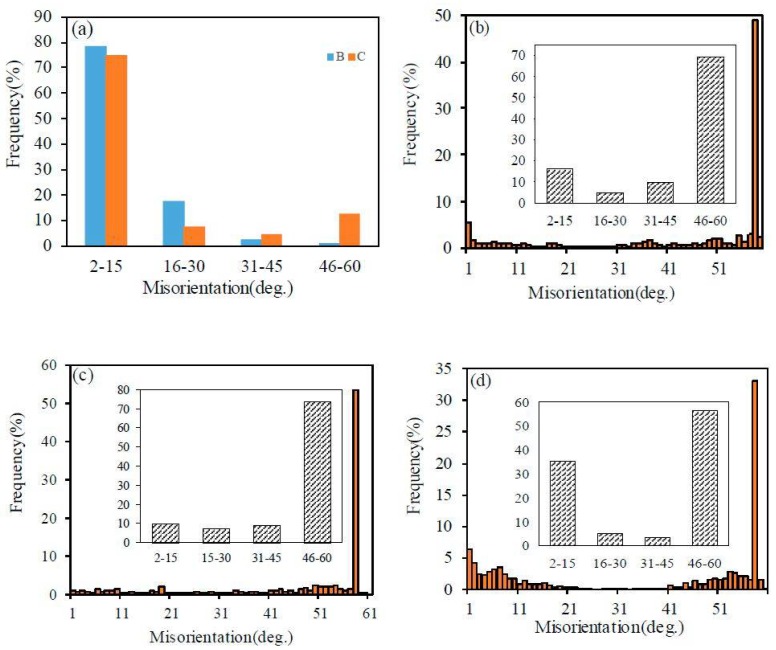
Misorientation distribution functions (MDFs) in α (**a**) of samples B and C, and in γ ((**b**)—A, (**c**)—B and (**d**)—C)).

Figure 5 shows the grain boundary misorientation distribution of α matrix and precipitated γ in samples A, B, and C. Most of the grain boundaries in α are the low-angle grain boundaries (LAGBs) in samples B and C. It is assumed that a number of fine sub-grains or grains interfaced by LAGBs was produced as the coarse original ferritic grains were largely strained and subsequently annealed. This process is associated with recovery in samples B and C during annealing [13,17]. LAGBs might serve as the nucleation site and therefore promote γ precipitation. A relatively uniform microstructure is achieved as compared to sample A.

The big difference in the GBCD lies in the fractions of LAGBs and TBs in γ for samples A, B, and C, as shown in Figure 5b–d. It can be seen that the sum fractions of TBs and LAGBs are nearly the same for all samples, at approximately 65%. However, the relative proportions of TBs and LAGBs are quite different, corresponding to 49% and 16% in sample A, 57% and 9% in sample B, and 35% and 32% in sample C, respectively. It is well understood that TBs are prevalent in γ due to the low stacking fault energy during annealing, and the fraction of TBs is typically 40–50% in a recrystallized single austenitic steel [18,19]. However, the large variation in the density of TBs and LAGBs also caused by the rolling mode (direct and cross rolling) is an interesting phenomenon and deserves a further explanation. After direct rolling and annealing, the precipitated γ grains tend to cluster and readily increase, as shown in Figure 3b. The process is similar to recrystallization and grain growth in single austenitic steel due to the absence of PBs in the local region, during which many annealing twins are formed. By comparison, the amount of TBs decreases significantly when cross-rolling and annealing are applied in sample C, as shown in Figure 3c’ and Figure 5d. The strain anisotropy is reduced and homogenous residual strains bring relative homogeneous precipitation. Therefore, no remarkable clustering in γ grains is observed in sample C. It should be noted that some γ grain clusters are found in a very small area in sample C, as evidenced in Figure 3d’. However, the TBs are still scarce. Hence, the formation of TBs might be restricted by other factors, which will be clarified in next section. As to the increase of LAGBs in sample C, it might be the result of sympathetic nucleation of γ precipitation [20,21] instead of twining. Twinning tends to randomize the orientation texture while LAGBs sharpen it. Sympathetic nucleation (SN) is defined as the nucleation of a precipitate crystal at an interphase boundary of a crystal of the same phase when these crystals differ in composition from their matrix phase throughout the transformation process. Hence, the results of the current microanalysis confirmed the occurrence of SN in this type of DSS.

### 3.3. Grain Orientation and Interfaces after TMP

Texture evolution has been extensively investigated in rolled and annealed DSSs. It was found that the resulting texture components mainly depends on initial microstructure [13], processing history [4,15], and phase transformation. This section primarily focuses on the orientation evolution of γ grains precipitated from the given α matrix based on the microtexture analysis.

**Figure 6:**
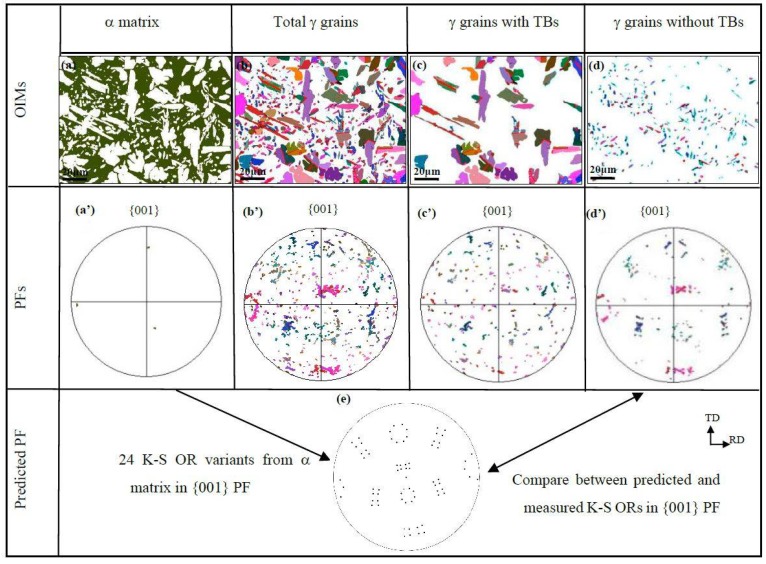
EBSD-reconstructed OIMs and {001} pole figures in sample A: (**a**–**d**) OIM maps; (**a’**–**d’**) measured {001} PFs; (**e**) predicted {001} PF of 24 K-S OR variants.

**Figure 7:**
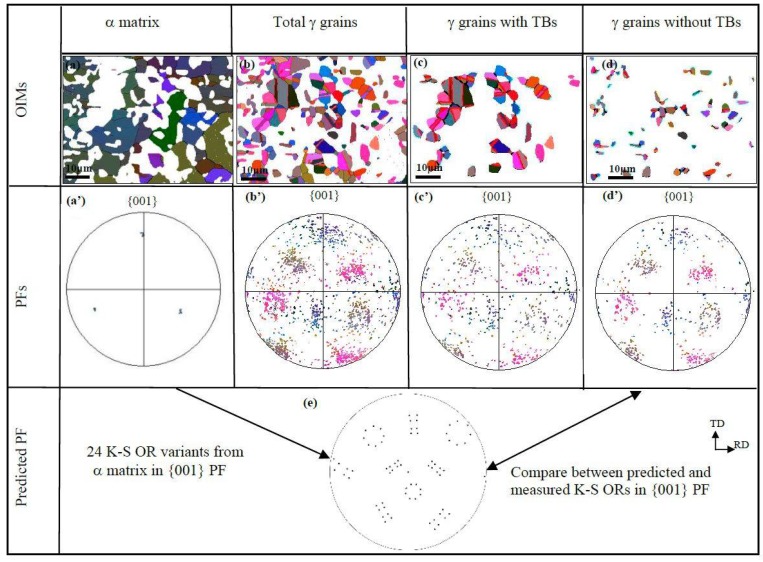
EBSD-reconstructed OIMs and {001} pole figures in sample B: (**a**–**d**) OIM maps; (**a’**–**d’**) Measured {001} PFs; (**e**) Predicted {001} PF of 24 K-S OR variants.

**Figure 8:**
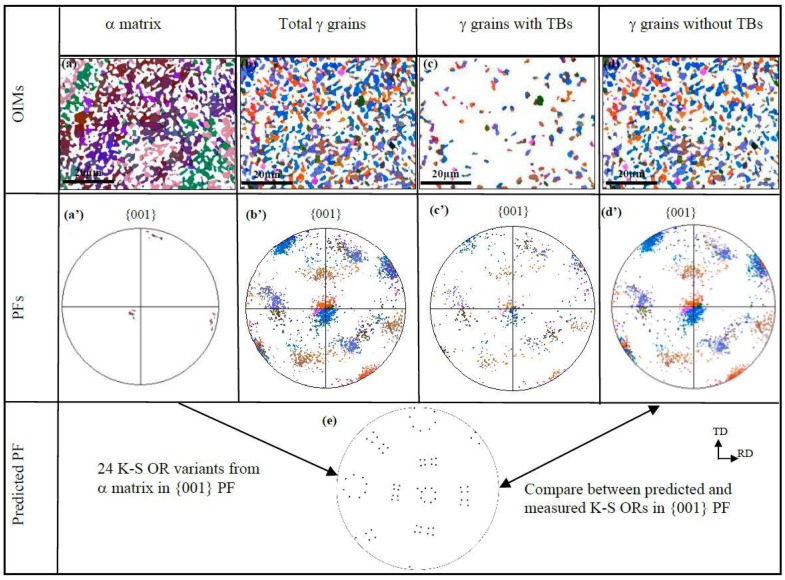
EBSD-reconstructed OIMs and {001} pole figures in sample C: (**a**–**d**) OIM maps; (**a’**–**d’**) measured {001} PFs; (**e**) predicted {001} PF of 24 K-S OR variants.

The grain morphology and {001} pole figure (PF) maps for the α matrix and precipitated γ in the sample A are separated and represented in the OIM maps (Figure 6a,b) and {001} pole figures (PFs) (Figure 6a’,b’). In the meanwhile, the precipitated γ grains are further divided into two subsets. One contains twin boundaries (TBs) within γ grains, while the other one does not. The corresponding OIMs and {001} PFs are also shown in Figure 6c,c’,d,d’, respectively. Similar microstructural information is also organized and represented in Figure 7 and Figure 8 for samples B and C, respectively.

For sample A, the orientation of α matrix is (012) $\left[\bar{1}\bar{6}3\right]$ and the precipitated γ grains have a spread orientation distribution in the {001} PF space. The phenomena become more pronounced when solely γ grains with TBs were analyzed, as shown in Figure 6c,c’. However, the orientation distribution tends to become regular and nearly identical to the predicted {001} PF pattern of the 24 exact K-S OR variants, as given in Figure 6e. It is obvious that the 24 K-S OR variants are nearly equally introduced.

The orientation of the α matrix in sample B is similar to that in sample A. They are disorientated by 5.5°/$\left[\bar{3}23\right]$. However, the precipitated γ grain orientation distribution was different. A slightly concentrated orientation distribution resulted in more precipitated γ grains as compared to sample A, which indicates that the pre-deformation of direct rolling exerted on the supersaturated α matrix has an orientation-strengthening effect on the precipitated γ grains. When solely the γ grains containing TBs were analyzed, the orientation concentration tended to weaken, as shown in Figure 7c,c’, confirming that twinning randomizes the texture of γ grains [22]. In addition, the γ grains containing no TBs usually held the K-S OR with the α matrix (Figure 2), but their orientation distribution in {001} pole figure (PF) indicated that no regular pattern of 24 K-S orientation variants (Figure 7d’) occurred, in contrast to the predicted {001} PF (Figure 7e).

As for sample C, a quite different orientation distribution was generated in the precipitated γ grains, as shown in Figure 8. The orientation of α matrix is selected as near a (001) [010] cubic type in the current study, as shown in Figure 8a’. The orientations of the precipitated γ grains were remarkably strengthened after cross-rolling and annealing. There are three main grain orientations, i.e., (203) $\left[0\bar{1}0\right]$, (012) [100], and (123) $\left[\bar{1}2\bar{1}\right]$, corresponding to the blue, orange, and brown grains in Figure 8b, respectively. It should be noted that the blue and orange grains are disorientated by 9.2°, indicating that the neighboring blue and orange grains can be related by LAGBs, contributing significantly to the density of LAGBs in the γ phase of sample C. It should be noted that the orientation distribution for γ grains with or without TBs do not exhibit much change, as shown in Figure 8c’,d’, suggesting that orientation randomization via twinning was not as pronounced in sample C as compared to samples A and B. This might be because twinning has a very limited effect on the orientation modification when γ grains have not undergone sufficient growth.

As observed in Figure 3, the γ grains containing no TBs show a tendency to satisfy K-S OR with their α matrix, especially in samples A and B. Thus, a question arises: is twinning restricted by the phase boundary with K-S ORs, or will it make the precipitated γ grains deviate from the K-S OR with the α matrix? Considering that more TBs were found within the larger γ grains (as shown in Figure 3, Figure 6, Figure 7 and Figure 8), twinning is prone to occur during grain growth, during which there might be orientation modification to some extent in both the parent and twinned γ grains. As a result, the γ grains no longer satisfy the K-S OR. By comparison, the γ grains with no twins typically follow a K-S OR with the α matrix, as shown in Figure 3a–c. If the measured {001} PF (Figure 7d’ and Figure 8d’) of the γ grains without twins and the predicted {001} PF (Figure 7e and Figure 8e) from the 24 exact K-S OR variants are compared, it can be observed that not all 24 variants are equally produced in the cold-rolled and annealed samples B and C. This means that variant selection occurred, especially in sample C, which contributes significantly to the strong texture.

**Figure 9:**
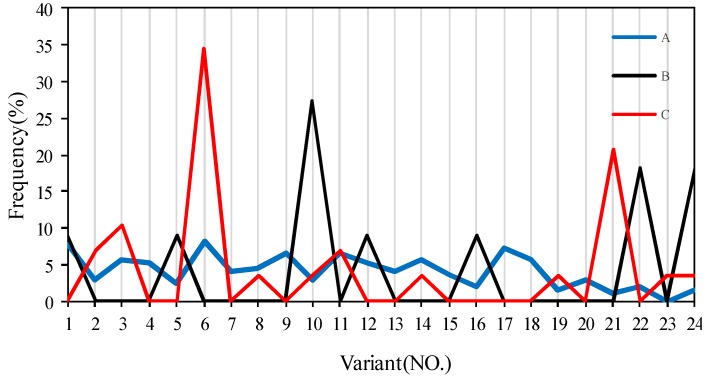
fraction of the 24 variants of the phase boundaries (PBs) with K-S ORs within the angular tolerance of 3.5°.

In order to determine the occurrence of variant selection, the OR matrix between each γ grain and its α matrix was calculated and then compared with the exact 24 K-S OR matrix. The misorientation between each two orientation matrices can be expressed in the form of angle/axis pairs (θ/<uvw>) [23,24,25]. Hence, a given γ grain can be identified as one of 24 variants within a certain angle tolerance. The number fraction of 24 variants within 3.5° is shown in Figure 9 for samples A, B, and C. It can be seen that 24 K-S variants are nearly uniformly distributed and no obvious variant selection was found in sample A. However, variant selection is remarkable in the cold-rolled and annealed samples, in particular in sample C. Variants 10 (v10), 22 (v22) and 24 (v24) in sample B and variants 6 (v6) and 19 (v19) in sample C were selectively introduced during annealing. The misorientation between each two variants could be calculated based on the orientation matrix, with values of 57.21°/$\left[\bar{3}56\right]$, 47.11°/$\left[\bar{24}\;\bar{10}\;21\right]$, 60°/[011] and 10.53°/$\left[11\bar{1}\right]$ for variant pairs v10/v22, v10/v24, v22/v24, and v6/v19, respectively. It should be noted that v6 and v19 are probably neighbored, and therefore a large number of LAGBs might have been produced in sample C, sharpening the orientation texture of the γ grains. Although strong variant selection occurs and a strong texture should be expected, extensive twinning weakened the texture strength in sample B. The competition of LAGBs and TBs within γ grains is a very interesting phenomenon. The formation of LAGBs within γ grains does not destroy the K-S OR, whereas the introduction of TBs typically does. It is assumed that the grain pairs with similar orientation might not have hard impingement due to the high frequency of LAGBs. The sympathetic nucleation proposed by Aaronson and Wells [21] should be responsible for the formation of the prevalence of LAGBs in sample C. The prior γ precipitation acts as the favorable nucleation sites for the later γ precipitation with similar orientation instead of continuous growth of prior γ precipitation. Obviously, this process is significantly strengthened by cross-rolling subjected to a supersaturated solid solution. It is suggested that the orientation of γ precipitation nucleus strongly depends on the magnitude and distribution of residual strain introduced by prior deformation in supersaturated ferrite. The inhomogeneous strain caused by direct rolling promotes clustering and twinning for γ grains in sample B, whereas the relatively uniform strain produced by cross-rolling tends towards sympathetic nucleation during annealing. The underlying mechanism remains to be further investigated.

## 4. Summary

Duplex stainless steels (UNS S32304) after solid solution treatment for 30 min at 1573 K were subjected to direct rolling and cross rolling with a true strain of ε = 2 and subsequent annealing for 2 min at 1323 K. The orientation of precipitation γ grains and the interface character distribution in the rolled and annealed samples were investigated along with the sample without pre-deformation using EBSD technology. The key findings are summarized as follows:

1. A more homogeneous and refined microstructure was obtained in the cross-rolled and annealed sample than in the direct rolled and annealed sample, in which γ grains were prone to clustering.
2. TBs were frequently found within γ grains precipitated from the direct-rolled supersaturated α due to the absence of phase boundary in the **γ** grain-clustered region during annealing. LAGBs and TBs coexist within γ precipitation from the cross-rolled supersaturated α due to the sympathetic nucleation during annealing.
3. The formation of TBs leads the γ grain orientation to be random and deviate from K-S OR, whereas the presence of LAGBs intensifies the γ grain orientation, maintaining the K-S OR with the α matrix.
4. Variant selection tends to occur more often in the rolled and annealed samples as compared to the directly annealed sample without pre-deformation. Variants 6 and 19 are frequently produced and are related by LAGBs in the cross-rolled and annealed samples.
